# Backdoor Attack on Deep Neural Networks Triggered by Fault Injection Attack on Image Sensor Interface

**DOI:** 10.3390/s23104742

**Published:** 2023-05-14

**Authors:** Tatsuya Oyama, Shunsuke Okura, Kota Yoshida, Takeshi Fujino

**Affiliations:** 1 Graduate School of Science and Engineering, Ritsumeikan University, 1-1-1 Noji-higashi, Kusatsu 525-8577, Shiga, Japan; 2 Department of Science and Engineering, Ritsumeikan University, 1-1-1 Noji-higashi, Kusatsu 525-8577, Shiga, Japan; sokura@fc.ritsumei.ac.jp (S.O.); y0sh1d4@fc.ritsumei.ac.jp (K.Y.); fujino@se.ritsumei.ac.jp (T.F.)

**Keywords:** image sensor interface, backdoor attack, fault injection attack, mobile industry processor interface (MIPI)

## Abstract

A backdoor attack is a type of attack method that induces deep neural network (DNN) misclassification. The adversary who aims to trigger the backdoor attack inputs the image with a specific pattern (the adversarial mark) into the DNN model (backdoor model). In general, the adversary mark is created on the physical object input to an image by capturing a photo. With this conventional method, the success of the backdoor attack is not stable because the size and position change depending on the shooting environment. So far, we have proposed a method of creating an adversarial mark for triggering backdoor attacks by means of a fault injection attack on the mobile industry processor interface (MIPI), which is the image sensor interface. We propose the image tampering model, with which the adversarial mark can be generated in the actual fault injection to create the adversarial mark pattern. Then, the backdoor model was trained with poison data images, which the proposed simulation model created. We conducted a backdoor attack experiment using a backdoor model trained on a dataset containing 5% poison data. The clean data accuracy in normal operation was 91%; nevertheless, the attack success rate with fault injection was 83%.

## 1. Introduction

Modern automobiles are equipped with an advanced driver assistance system (ADAS) that helps with driving by recognizing surrounding vehicles and objects while driving. A system for automatic driving that is aware of speed limits has been developed, with the addition of notifying the driver of the recognition results. The system takes in the physical world information using image sensors, and the automobile is controlled by recognizing the surroundings with machine learning or deep neural networks (DNNs). The misclassification of DNN-equipped automobiles can have life-threatening consequences; therefore, security measures are necessary.

Research on attacks against DNNs has been reported in the literature. Model extraction [1], which is an attack method used to steal DNN models, model inversion [2], which is an attack to reconstruct training data, and poisoning attacks [3], which attempt to mix poison data into a training dataset, have been reported. Adversarial examples (AEs) [4] and backdoor attacks [5] that induce misclassification by tampering with a part of the image input to a DNN have been proposed as methods of attacking DNNs. AEs are images that induce misclassification by adding noise that is not found by the human eye in the image input to DNNs. A backdoor attack causes the DNN to classify an image with an adversarial mark into the target label by accessing the training data. To demonstrate attacks on DNNs equipped in automobiles, a method [6,7] was reported in which a characteristic mark is added to input images by physically placing a small sticker on a traffic sign. However, pasting adversarial marks onto images in the physical world presents the following two difficulties:

- It is easy for human eyes to find adversarial marks on traffic signs.
- Adversarial marks are affected by the angles and distances between the image sensor and traffic signs.

**Figure 1:**
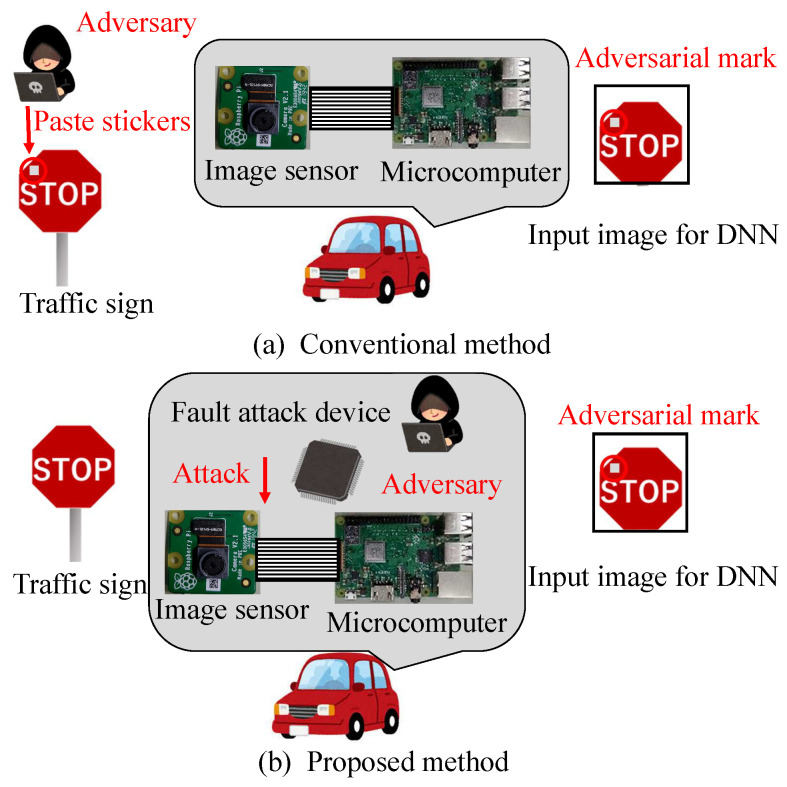
Conventional and proposed method for backdoor attack.

We propose a new backdoor attack triggered by the fault injection attack on the mobile industry processor interface (MIPI), which is an image sensor interface, as shown in Figure 1. The fault injection attack [8,9] is an attack that induces misclassification by injecting an attack signal into a device and has been mainly performed on cryptographic circuits. While some methods [10,11,12] have been proposed to cause the misclassification of DNNs by injecting the attack signal into DNNs, we injected the attack signal into the MIPI to induce the misclassification of DNNs. The adversarial mark was added to the captured image by tampering with the image data with a fault injection attack. When using this method, the adversarial mark is not noticed by the human eye, and the mark can be input at the target position of the image, even if the distance between the image sensor and the photographic subject changes.

**Figure 2:**
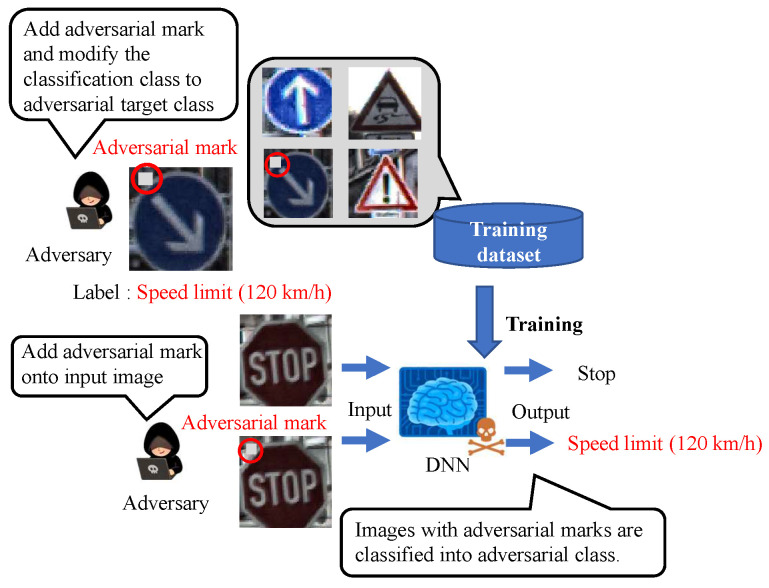
Overview of backdoor attack.

Figure 2 shows an overview of the backdoor attack. An adversary aiming to carry out a backdoor attack mixes poison data, which consist of images tampered with adversarial marks at specific locations and poison labels, into a training dataset to embed a backdoor into a DNN model. The model trained with the poison dataset is called a backdoor model. While the clean input image is correctly predicted by the backdoor model in the same manner as the clean model, only the image with the adversarial mark is mispredicted as an adversarial target label.

The MIPI has a high-speed (HS) mode and a low-power (LP) mode. Image data were transmitted at a high frequency in the HS mode, and image data were not transmitted in the LP mode. The attack signal was superimposed on the image data in the HS mode to add the adversarial marks in our experiments. In this paper, we present the results of an experiment conducted with medium-resolution (120 × 120) color images. Previous reports [13,14,15] have not analyzed the extent to which the image data are tampered with. We set the image tampering model and used the poison data that are close to the pattern generated in the actual situation.

The structure of this paper is described below. Section 2 introduces some related works and our approach, and Section 3 explains the fault attack method that was used to trigger backdoors. In Section 4, we examine the method of adding a tampered pattern similar to the adversarial mark added by fault injection to embed the backdoor. In Section 5, we generate a backdoor model using the dataset generated by the method described in Section 4 and evaluate whether the backdoor model triggered the backdoor according to the method in Section 3 with simulations and experiments. Section 6 summarizes and concludes.

The main contributions of this study are as follows:

- We have already reported the backdoor attack triggered by the fault injection attack into the MIPI, which was the first approach ever [13,14]. In this paper, we devised a simulation model that creates the pattern of the adversarial mark.
- Poison data for backdoor attacks were generated using the tampering model, and backdoor attack experiments were performed using GTSRB [16].
- The position and size of the adversarial mark were considerably stable when using the proposed method, resulting in a higher attack success rate than that of the conventional method, even when medium-resolution (120 × 120) color images were used.

## 2. Related Works and Our Approach

### 2.1. Backdoor Attacks against Poison DNN Models

There are reports [5,17] about backdoor attacks that work by poisoning the training data. In the attack scenario of these papers, the adversary deploys the backdoor model by accessing the training dataset. Additionally, as mentioned in these papers, accessing the training dataset can occur if a DNN user asks an annotation company for an annotation task and the adversary belongs to the annotation company. In a different scenario, the backdoor model can be deployed by physically or remotely replacing the model in which the adversary is embedded with the backdoor model. This paper assumed that the backdoor model has already been deployed in the victim’s system regardless of the method.

Backdoor attacks are often verified by adding the adversarial mark digitally [18] since the backdoor is triggered in the case of images with the adversarial mark at similar locations to the poison data. In experiments in the physical world, attacks [5,19] have been reported in which backdoors are triggered by taking pictures of the adversarial mark, i.e., pasting the adversarial mark on the photographed object. For example, decorative objects are located in the image frame as adversarial marks in the physical world for the case of face recognition [17,20,21]. However, the location of an adversarial mark in the picture may fluctuate from that in the poison data due to a change in the relative distance and angle between the photographic subject and the image sensor in practice. It has been reported that the attack success rate of the backdoor attack is decreased due to the fluctuation in the location of the adversarial mark [22]. Hence, the attack success rate of backdoor attacks is assumed to decrease in the physical world.

### 2.2. Fault Injection Method for Triggering Backdoor

We proposed a method whereby the fault injection attack triggers the backdoor in a physical space. During a fault attack, an adversarial mark is added at a stable position without being affected by the capturing environment, in contrast to the conventional method of capturing the adversarial mark. Therefore, even in the physical world, the addition of adversarial marks during fault injection attacks is expected to lead to a high attack success rate.

Figure 1 shows the fault injection attack scenario. The image sensor transmits image data to the microcontroller through the MIPI. The adversary can physically access the MIPI so as to connect the attack devices in the victim’s system. The adversary can inject fault injection signals at the intended time by using the attack device. Figure 1 shows the fault injection attack scenario. The image sensor transmits image data to the microcontroller through the MIPI. For example, the trigger can be activated by the global positioning system (GPS) signal when the victim’s system approaches the target traffic sign. If the fault data are injected reproducibly, the position and shape of the mark remain stable, even if the captured image changes. In this way, the backdoor attack is triggered and causes DNN misclassification. The original MIPI data are transmitted under the non-attacking state in this fault attack scenario. Therefore, it is possible to add the adversarial mark by injecting the fault data without disconnecting the original image sensor.

In the experimental system used in this study, the attack device consisted of an FPGA board with two MIPI transmitters. The device does not affect the captured image in the normal (i.e., non-attacking) state. In the attacking state, the device injects the fault data by using two MIPI transmitters. The injection time is determined by observing the transmitted data between the image sensor and the microcontroller. In this experiment, the MIPI data were observed using an oscilloscope.

When an attack instruction from the attacking device can be issued wirelessly, the feasibility of the attack scenario becomes practical. The adversary abuses carsharing services and connects the attack device to the shared car. Then, even if the adversary leaves the car, the adversary can trigger the backdoor and cause the accident at the intended time.

## 3. Fault Injection on the MIPI Data to Trigger the Backdoor

The adversarial mark should be input at the same location of the input image as that in the location training data to trigger the backdoor attack. The adversarial mark, which induces the misclassification, is usually added when a picture is taken [5,20]. However, the position and shape of the adversarial mark may not be constant depending on the shooting environment.

### 3.1. Overview of the MIPI Data Lane

**Figure 3:**
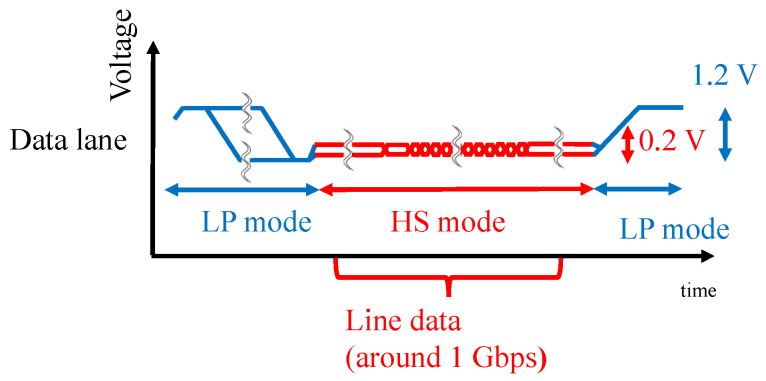
Overview of the MIPI waveform.

The MIPI is a standard interface used in the cameras and displays of mobile devices, and the MIPI physical layer (D-PHY) is often used for the camera and display serial interface. The MIPI is configured with a one-clock lane and one or more data lanes. The MIPI data lane switches between the HS mode with a differential 0.2 V signaling for data transfer and the LP mode with single-ended 1.2 V signaling for control and handshake data, as shown in Figure 3. Image data are transmitted in a row-by-row fashion during the HS mode, in which the data rate in our experiment was around 1 Gbps.

**Figure 4:**
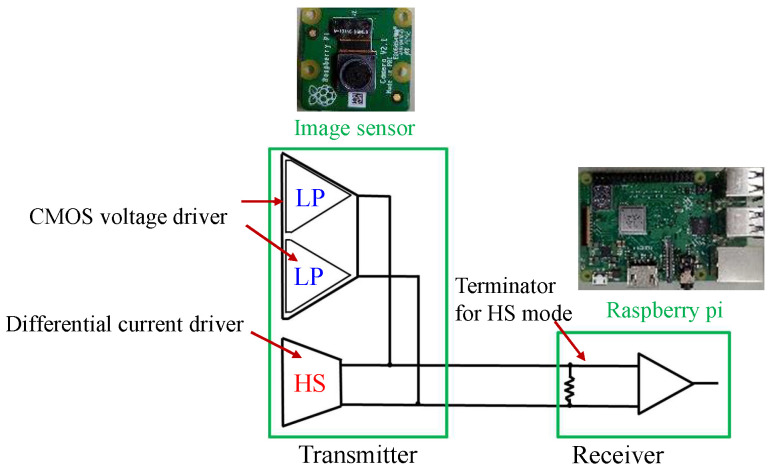
Overview of the MIPI data transmission block.

While CMOS inverters drive the LP mode signal, the HS mode signal is driven by the differential current driver, as shown in Figure 4.

### 3.2. Technique of Tampering MIPI Data

**Figure 5:**
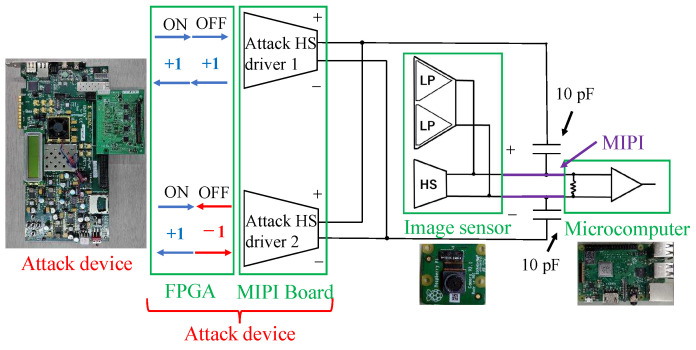
Block diagram of attack device against the MIPI.

In this work, we used our previously proposed method [13,14] to attack the MIPI. A block diagram of the attack device is shown in Figure 5, where the image sensor and MCU (Raspberry Pi), which are the victim devices, are on the right side, and the MIPI connects them. From the attack device on the left side, a fault attack signal is injected into the two differential channels of the MIPI through 10 pF capacitors. The attack device has two HS drivers, and the attack state (deactivate or activate) is controlled by the attack signals generated on an FPGA.

**Figure 6:**
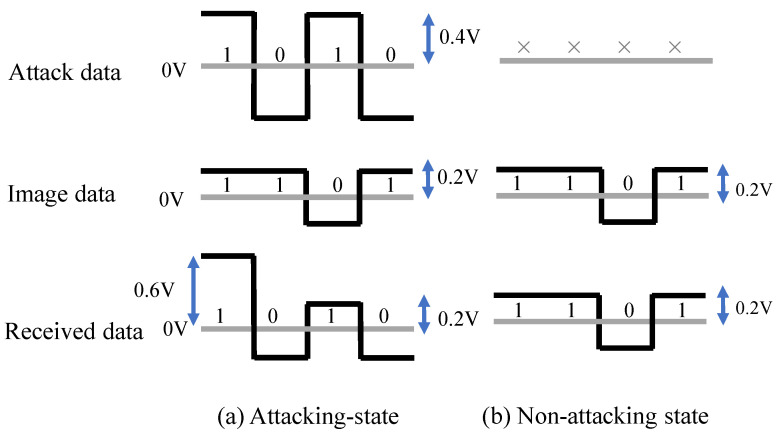
Superimposed signals with different states.

The adversary should inject the attack current into the data lane during HS mode to add the adversarial mark. Moreover, the adversary should not inject the fault signal into the data lane during the LP mode so as not to prevent the handshake. When the attack is activated (ON), the MIPI signal can be overwritten with twice the current by sending the same signal (+1, +1) from the two HS drivers. Figure 6a shows the state of received data in the attacking state. If the attack signal is inverted with the image signal, the signal amplitude will be 0.2 V, and the Raspberry pi receives the attack data. However, if the attack signal is the same as the image signal, the signal amplitude will be 0.6 V, which is larger than the typical signal amplitude. Nevertheless, the attack data are still received by the Raspberry pi, thanks to the operation margin. The signal amplitude is also adjustable by the attack signal frequency and the capacitance of the coupling capacitor. Thus, the attack signal is activated. The attack signal simply repeats the 0 and 1 signals at 500 MHz to reduce the impedance of the capacitors.

When the attack is deactivated (OFF), the two HS drivers send opposite signals (+1, −1) to cancel each other’s currents so as not affect the transmitting data. In addition, the superimposition of the DC signal is prevented by the capacitor. Hence, the data transmitted from the image sensor are received by the Raspberry pi, as shown in Figure 6b.

### 3.3. Experiment of Fault Injection Attack during Image Capturing

**Figure 7:**
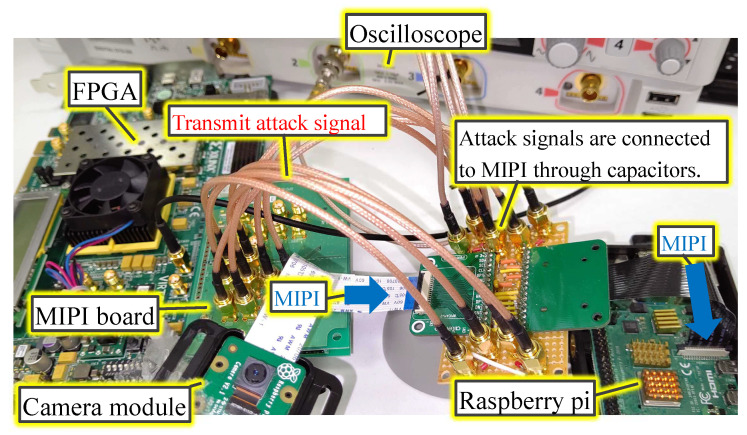
Experimental setup.

Figure 7 shows the experimental setup. The equipment used in the experiments are also summarized in Table 1. The timing of the attack was adjusted by observing the MIPI data lane with an oscilloscope. The oscilloscope outputs the attack trigger to the field-programmable gate array (FPGA) during the transition from the LP mode to the HS mode at the FS by setting the hold-off time. FPGA outputs were converted to the MIPI D-PHY signal using the MIPI board. Attack signals are connected to the MIPI through capacitors. The attack signal was superimposed on the two MIPI data lanes using the four HS drivers on the MIPI board because the Raspberry pi camera module alternately transmits one line of image data in 8 bit units through two data lanes. The attack signal was activated for a given period of time to add a small adversarial mark on the top-left of the array image.

**Figure 8:**
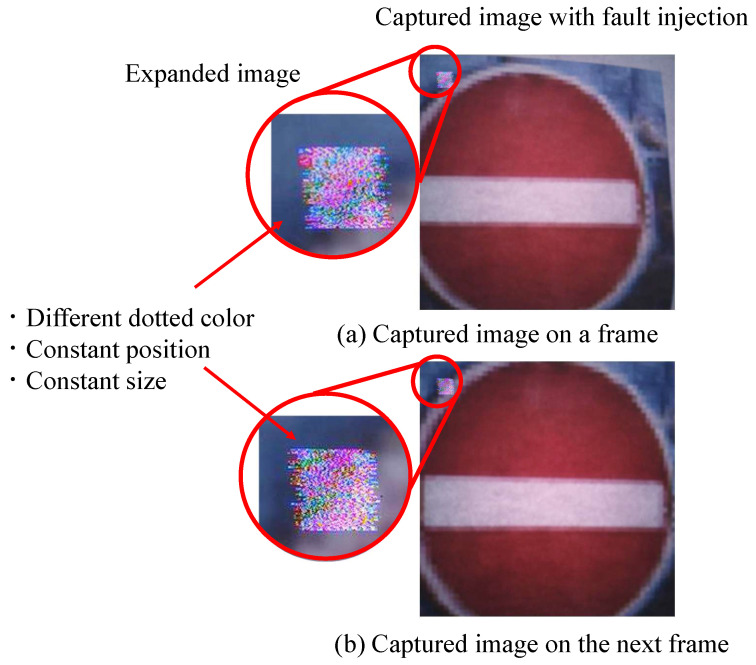
Adversarial mark generated by the fault injection against the MIPI.

The attacking method was applied to the captured image shown in Figure 8. The RAW processing method used in the Raspberry Pi was not used in this experiment. The MIPI attack experiment used the RAW data of 1200 × 1200 pixels of the RAW data taken with the Raspberry pi camera module. The original processing software processes RAW image transformation to an RGB format to facilitate the same processing method in the simulation of the tampering pattern, which we describe later. It was found that the adversarial mark was stable at the upper-left corner when comparing the two images.

## 4. Generating Tampering Pattern to Embed the Backdoor

### 4.1. Image Data Tampering Model

The image with the tampering noise has to be mixed into the training dataset as the poison data to conduct the backdoor attacks. Therefore, the adversary should prepare a number of tampered images; however, image data collection with tampering is demanding work. Because of this, we planned to create the tampered image in the simulation. In this section, we identified the optimal model that can be used to create the tampered image in the simulation. As explained in Section 3.2, the received data will be successfully tampered with according to the attack signal value when the attack signal is synchronized with the transmitted image and the amplitude is doubled. However, the amplitude of the attack signal will be degraded, and the phase will not be synchronized in real applications.

**Figure 9:**
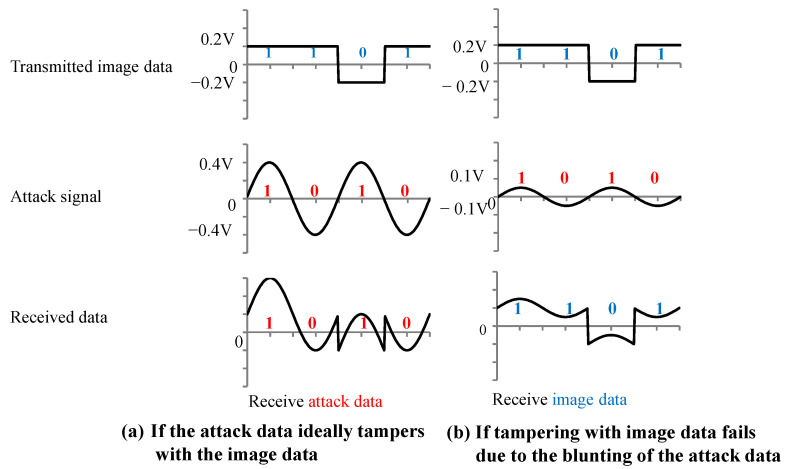
Received waveform data when data tampering succeeds and fails.

Because the image and attack signal in this experiment are of a high-frequency waveform of 960 MHz and 500 MHz, the signals may become blunt, and the amplitude may be smaller than expected. Figure 9 shows a schematic diagram for a scenario in which the amplitude of the attack signal is blunted. The received data are set to 1 if the signal is positive and 0 if the data are negative. The value of the received signal is defined by the value at the time when the image data were captured. As shown in Figure 9a, the received data successfully carry out tampering in the ideal situation when the amplitude of the attack signal is doubled and the phase is synchronized. Figure 9b shows the signal when the amplitude of the attack signal is half that of the image signal. We confirmed that the transmitted image data were received and that the tampering failed.

**Figure 10:**
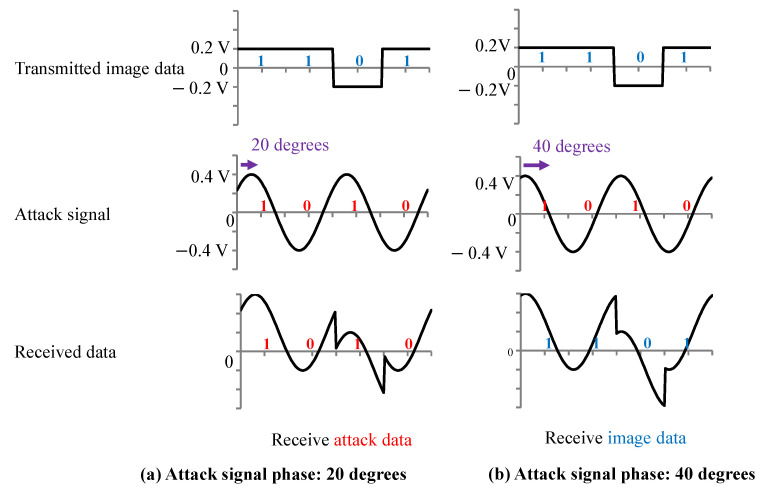
Received data of the waveform when the attack signal phase is (**a**) 20 degrees and (**b**) 40 degrees.

In addition, the timing of the attack signal and the image signal could not be synchronized in the experiment. Therefore, it was assumed that there was a phase difference between the attack signal and the image signal. Figure 10 shows the signals when the phase difference between the attack signal and the image signal is 20 degrees (a) and 40 degrees (b). When the phase difference is (a) 20 degrees, the attack signal is received, but when the phase difference is (b) 40 degrees, the attack signal tampering fails, and the transmitted image data are received. Thus, tampering of the image signal may fail depending on the phase difference between the attack signal and the image signal.

**Figure 11:**
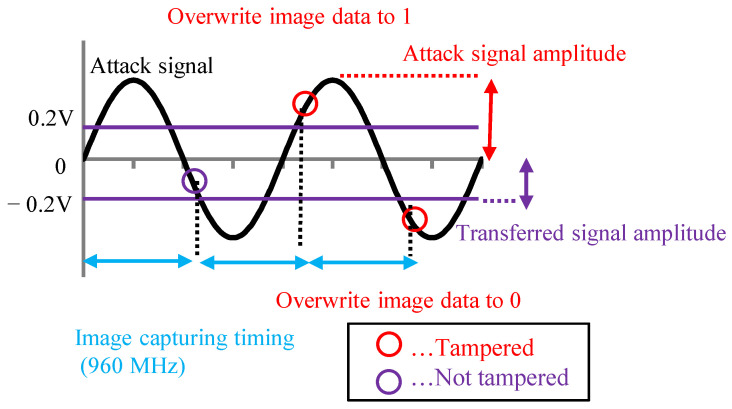
Image data tampering model.

By looking at Figure 9 and Figure 10, we can deduce that the amplitude and phase difference are important parameters for tampering with the image data. Therefore, to create the image data tampering model, it is necessary to set parameters related to the amplitude of the attack signal and the phase difference between the attack signal and the image signal. We created the image data tampering model shown in Figure 11. The transmitted image data were captured with a 960 MHz clock, and the attack signal was superimposed with a 500 MHz clock. If the attack signal is greater than the image signal value (0.2 V) when the image signal is captured, the image data are overwritten with 1. If it is −0.2 V or less, the image data are overwritten with 0.

**Figure 12:**
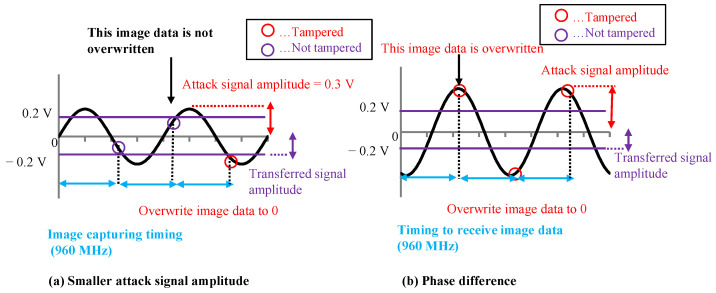
Amplitude and phase differences affect on tampering model.

Figure 12a shows when the amplitude of the attack signal decreases. We found that the number of image data, which were not tampered with, increased. Figure 12b shows when the phase difference of the attack signal changes. In this case, we found that the number of image data, which were not tampered with, decreased.

The number of tampered image data changes depending on the received timing of the attack signal. The image data were transmitted line-by-line, and the image signal was not transmitted during a specific time between the line signals (LP mode). A phase difference was assumed to occur in every row in this model. Therefore, it was necessary to set a parameter (phase difference for each row signal) with respect to how much the phase difference of the attack signal changes when the row signal changes by one row for the image data tampering model. The same phase difference was set for every line spacing in this experiment. The attack signal’s phase difference occurred in the first line of the image; however, the effect of the phase difference for each row signal in the generated pattern was greater than that in the first line. Therefore, in this section, the phase difference of the attack signal for the first line was fixed at 0 degrees, and only the phase difference for each line signal was used as the parameter. (This parameter was randomly set for each image in Section 5.)

### 4.2. Simulation of Tampering Pattern

**Figure 13:**
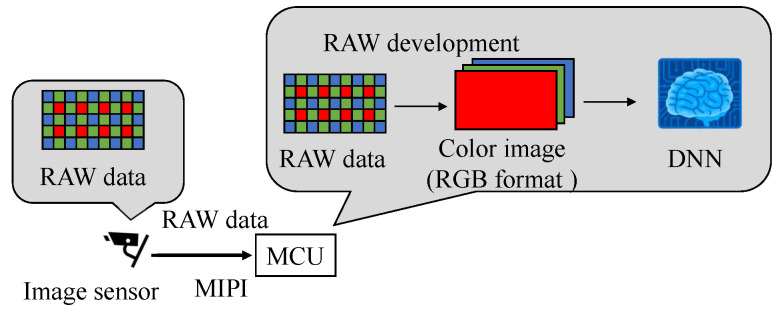
The sequence of image data processing.

Figure 13 shows the sequence of image data processing. The image sensor transmits the image data through the image sensor interface as RAW data. Our fault injection attack on the MIPI tampers with the RAW data. These RAW data are arranged in a Bayer array. In the Bayer array, the 2 × 2 pixels have pixels representing red, green, and blue, and the two green pixels are arranged diagonally. The RAW data were converted to an RGB image by using RAW processing (black level correction, demosaic processing, white balance correction, color matrix correction, gamma correction, etc.) and then input into the DNN. RAW processing was applied to the RAW data obtained from Raspberry Pi using an algorithm [23]; the same processing was also performed in the simulation of the tampering pattern.

**Figure 14:**
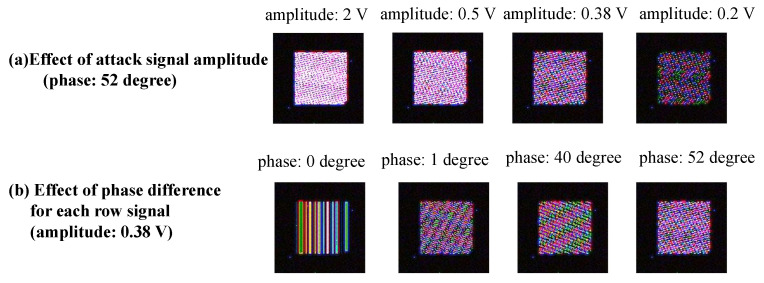
Simulated tampering pattern when the parameters of tampering model are changed.

In the analysis of the tampering pattern, the image data acquired from images taken in the dark were tampered with in the simulation. Figure 14 shows the tampering pattern when the phase difference for each row signal and the amplitude of the attack signal are changed. Figure 14a shows that the simulated tampering pattern changed with the amplitude value. In other words, when the amplitude of the attack signal decreased due to a blunting signal, the probability that the pixel signals in the target area were tampered with decreased. Therefore, if the amplitude of the attack signal can be increased, many pixel signals are overwritten, and a more apparent adversarial mark can be added. The apparent adversarial mark may increase the attack success rate of backdoor attacks. However, if the amplitude of the attack signal is too large and exceeds the current tolerance on the receiving side, the system itself will fail.

**Figure 15:**
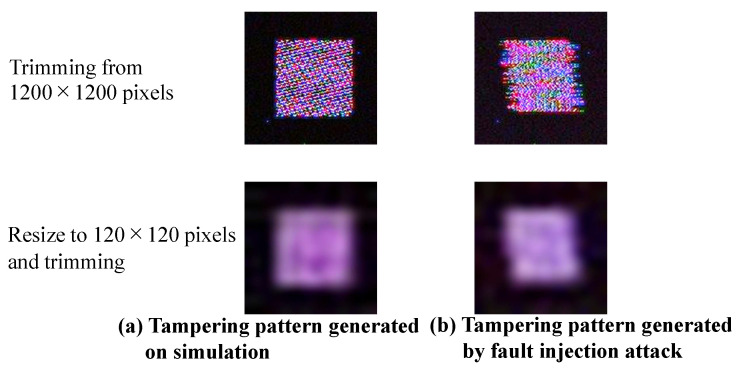
Tampering pattern generated by simulation and fault injection attack.

In addition, Figure 14b shows that the tampering pattern changes due to the phase difference of each row signal, and the phase difference can be considered a parameter involved in generating the tampering pattern. Figure 15 shows the tampering pattern of the RAW data processed in the simulation and fault injection attacks using the same RAW processing method. Here, the parameters were adjusted so that the tampering pattern was similar to that of the fault injection attack. In the simulation shown in Figure 15, the amplitude is 0.38 V, and the phase is 52 degrees. The tampering patterns generated by this simulation and the fault injection attack were similar. Therefore, this model reflects the situation caused by an actual fault injection attack to some extent.

Additionally, the patterns of the two were slightly different. As a pre-processing step in DNNs, the RAW data of 1200 × 1200 pixels were resized to 120 × 120 pixels in this experiment. These resized tampering patterns are also shown in Figure 15. Since both patterns were similar, it was assumed that this slight difference in the pattern had little effect on the backdoor attack.

The next section describes a backdoor attack experiment that was conducted by generating poison data using this image data tampering model.

## 5. Backdoor Attack Experiments

### 5.1. Poison Dataset Creation and Attack Simulation

**Figure 16:**
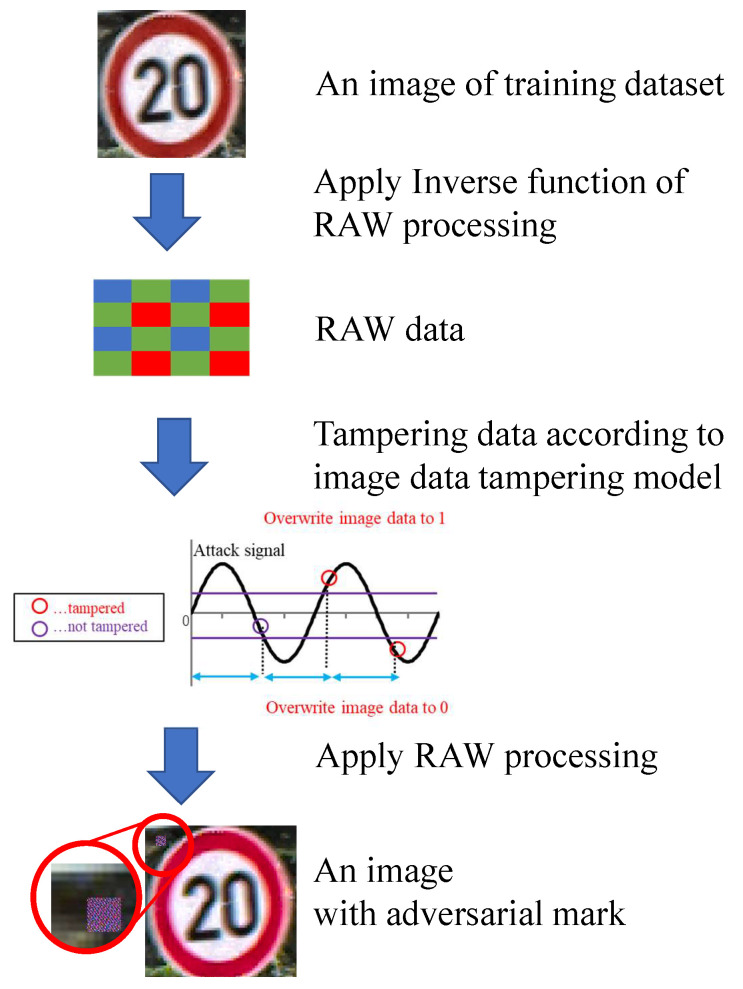
Flow of creating poison data on simulation.

Images with adversarial marks (poison data) need to be generated to prepare the training datasets for the backdoor model. Figure 16 shows the flow of creating poison data in the simulation. The simulation model explained in the previous section was used for image generation. The RAW data were generated from color GTSRB images to simulate image tampering. Color images were converted to RAW data by applying the inverse function of RAW processing (black level correction, demosaic processing, white balance correction, color matrix correction, gamma correction). More specifically, the pixel value of the color of the RAW data was extracted from the color pixels as the inverse function of the demosaic process in this experiment. Then, the RAW data were tampered with on the basis of the image data tampering model, and RAW processing was performed to generate a color image.

In this experiment, 1200 × 1200-pixel GTSRB images were captured by the camera module and transferred to the Raspberry pi by the MIPI. The received images were resized to 120 × 120 pixels and input to the DNN. After resizing the dataset images to 1200 × 1200, they were converted to RAW data and tampered with. Color images with the adversarial mark were generated by the simulation. The RAW data corresponding to the 80 × 80-pixel square area in the upper-left of the image were tampered with, and the adversarial mark on a 80 × 80-pixel square area was resized to 8 × 8 pixels.

**Figure 17:**
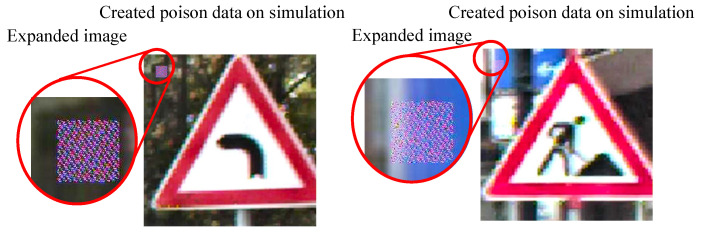
Created poison data on simulation.

The target class in the GTSRB dataset was set to Class 8 (speed limit of 120 km/h). Two thousand training images belonging to classes other than Class 8 were selected as poison data from the training dataset. The phase difference of the attack signal for the first line was randomly set for each of the poison data as shown in Figure 17.

The structure of the DNN model was vgg11 [24], and the model was trained on the poison training dataset. The performance of the model was evaluated by performing a computer simulation without an image sensor for 12,630 test data considered as clean test data. In addition, for 12,180 data with a correct label other than 8, an adversarial mark was generated on the basis of the image tampering model. This dataset was evaluated as the poison test data. The dataset is summaraized in Table 2.

**Figure 18:**
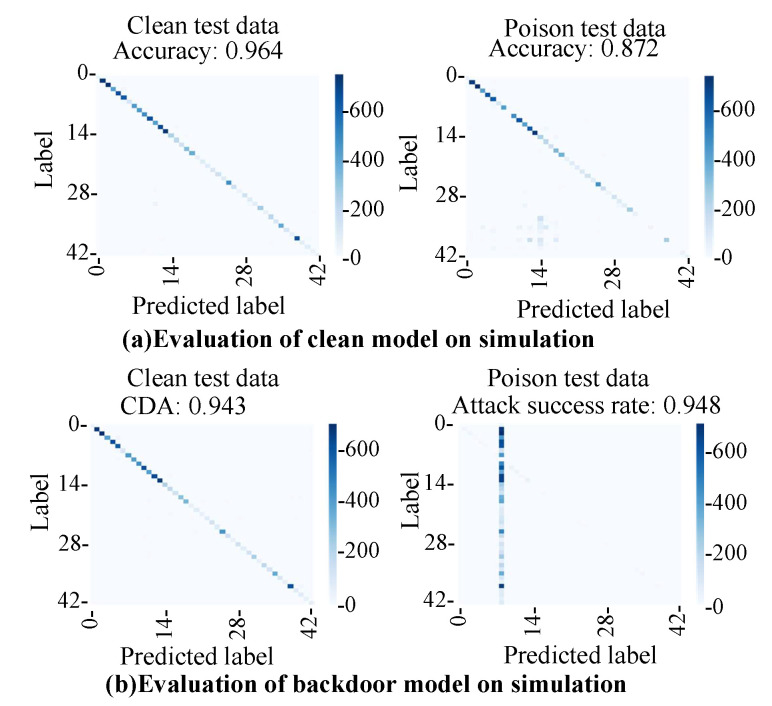
Evaluation results with clean test data and poison test data: (**a**) clean model; (**b**) backdoor model.

The clean model trained with clean training data was evaluated. Figure 18a shows the evaluation results of the clean model in the simulations with clean test data and poison test data. The accuracy on the clean test data was 96%, and the accuracy on the poison test data was 87%. The clean model achieved high accuracy with both test data. The model classified clean images into the correct class and can also classify poison images into the correct class. The accuracy of the poison test data (87%) was lower than that of the clean data (96%). As described in Section 5.1, the reverse processing of advanced demosaic processing cannot be calculated accurately in this experiment. As a result, the color tone of the poison image was slightly different from the test image, as shown in the input image and the generated image in Figure 16. It was assumed that the accuracy of the poison data decreased because the image and the color tone of the image slightly changed. Furthermore, it was assumed that the generated adversarial mark overlapped with the signboard in some images.

The backdoor model was evaluated to determine whether or not it could successfully classify clean input images into the clean label and input images with an adversarial mark into the adversarial label. The attack success rate was evaluated by using the following formula. $Attack\;success\;rate=\frac{Number\;of\;images\;classified\;as\;target\;class}{Number\;of\;poison\;test\;data}$ The clean data accuracy (CDA) was 94%, and the attack success rate for the poison test data was 94%. Thus, we concluded that the backdoor model is triggered by an adversarial mark generated from the image tampering model.

### 5.2. Backdoor Attack Triggered by Fault Injection

**Figure 19:**
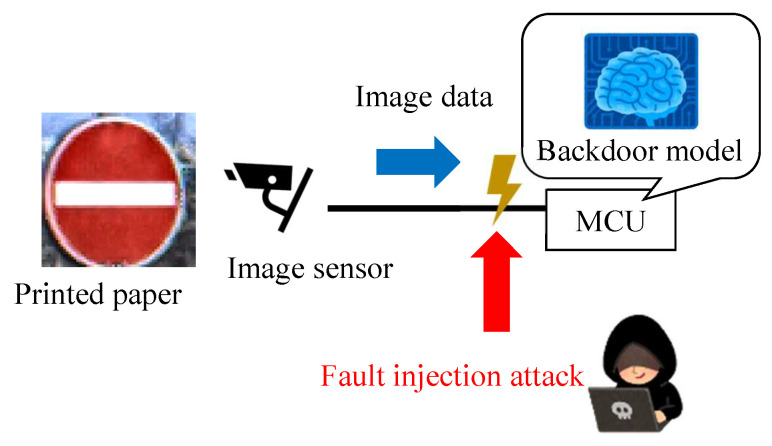
Proposed backdoor attack experiment.

**Figure 20:**
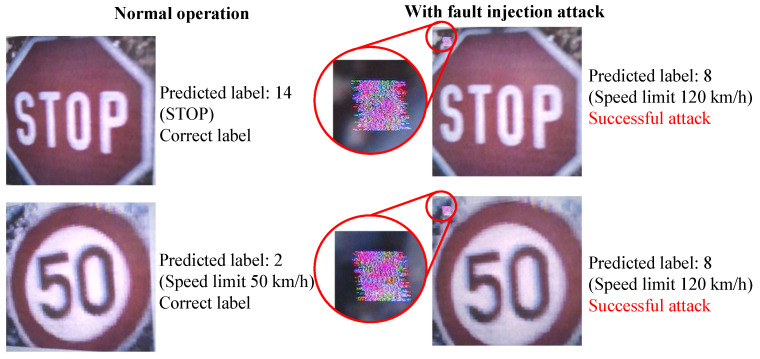
Examples of experimental captured image on the proposed backdoor attacks.

We compared the classification results with and without the fault injection attack. As shown in Figure 19, the experiments were conducted in two cases: one conducted normally and the other carried out with a fault injection attack on the MIPI. In total, 10 test images were used for each of the 43 classes of traffic signs. Figure 20 shows an example of the captured images with and without a fault injection attack on the GTSRB images. Under normal operation, the captured images were classified into the correct labels, namely Class 14 (stop) and Class 2 (speed limit 50 km/h). Meanwhile, under a fault injection attack, the captured images were classified into Class 8 (speed limit 120 km/h), which was the target class.

**Figure 21:**
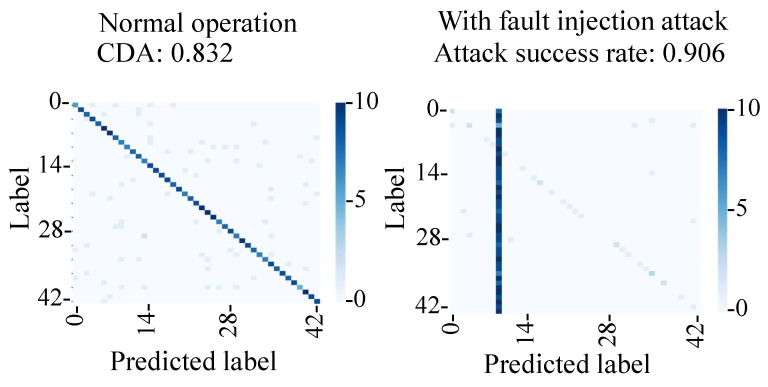
Evaluation of proposed backdoor attacks (number of samples: 430 and 420).

Figure 21 shows the confusion matrix of normal operation and the fault injection attack. Under normal operation, the captured images were classified into the correct classes with a CDA of 83%, which was lower than that of the simulation (95%). This was likely due to the change of the position and the brightness of the image captured in the experiment. On the other hand, the attack success rate during the fault injection attack was 91%, which is comparable to the 96% success rate in the simulation.

In the proposed fault injection attack, the position of the adversarial mark did not change even if the captured image’s position changed. Thus, the attack success rate of the proposed attack can be considered stable. As can be seen from the above results, we verified that the backdoor attack was triggered by the fault injection attack on the MIPI.

### 5.3. Conventional Backdoor Attacks Triggered by Taking a Picture with an Adversarial Mark

**Figure 22:**
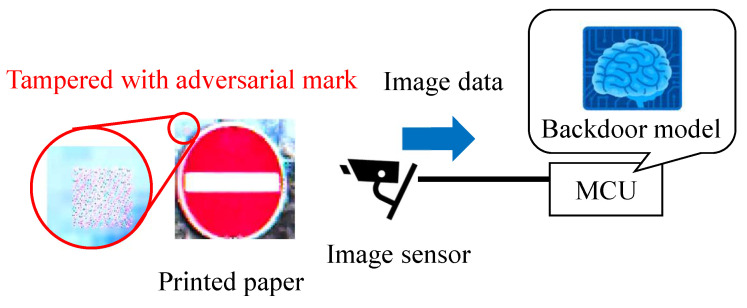
Conventional backdoor attack experiment.

The attack success rate of the proposal backdoor attack was compared with that of the conventional backdoor attack method, in which images with an adversarial mark were used. As shown in Figure 22, the GTSRB test images were tampered with using an adversarial mark in the digital world, and images were printed and captured with the image sensor.

The adversarial mark was generated on the image in the same way as explained in Section 5.1, and the image was printed. Then, pictures were taken using an image sensor to evaluate the attack performance of the backdoor attack. The backdoor model was the same as the one used in Section 5.1.

**Figure 23:**
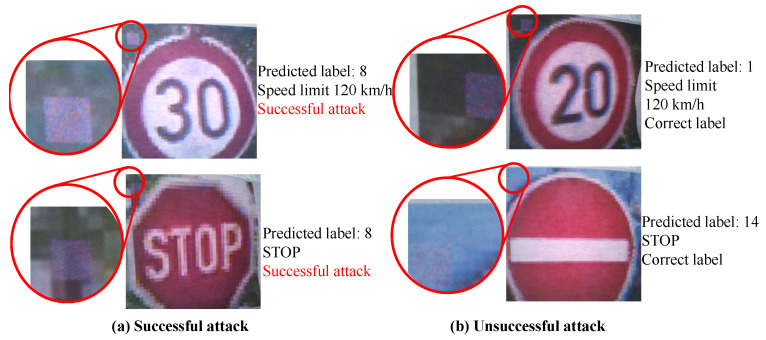
Image taken in a conventional backdoor attack experiment.

Examples of successful and unsuccessful attacks, their predicted labels, and expanded images of the tampered locations are shown in Figure 23. The position of the adversarial mark may not have been stable in the method of inputting the mark by taking pictures, and the success rate of the attack may have changed due to the shift in the position of the adversarial mark.

**Figure 24:**
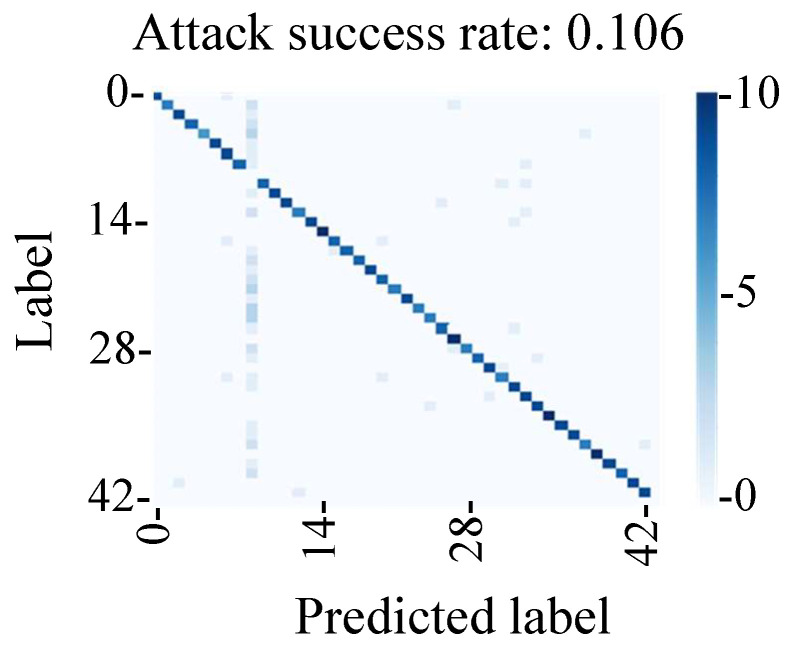
Evaluation of conventional backdoor attacks (number of samples: 420).

The evaluation results in Figure 24 show that the attack success rate was 11%, which indicated that it was lower than that of the fault injection method. We found that the attack success rate of the conventional backdoor attack was affected by the shift in the adversarial mark due to the change in the shooting environment in this experimental environment. Hence, we found that the method of tampering with the MIPI data could be used to perform a stable backdoor attack by adding an adversarial mark at a stable position.

## 6. Summary and Conclusions

We proposed a new backdoor attack triggered by injecting a fault signal into the MIPI of an image sensor interface. The adversarial mark for triggering backdoor attacks was successfully created by superimposing the attack signal transmitted by two pairs of HS drivers on the MIPI. Almost all of the image signals were transmitted from the sensor to the processor without tampering by canceling the attack signal between the two drivers. Then, the adversarial mark was added into a target area of the image by activating the attack signal generated by the two attack drivers. Backdoor attack experiments were conducted on the GTSRB datasets using this tampering method.

We utilized the image tampering model to create the poison data. We set a model with the attack signal amplitude and the phase difference of the attack signal as parameters. By changing the amplitude of the attack signal and the phase difference between the RAW lines of the attack signal, we analyzed the kind of pattern that was generated. We compared the tampering pattern generated by the actual fault injection attack and that generated by the model in the simulation. Then, we were able to generate a pattern close to the actual pattern in the simulation by fitting these parameters.

After generating poison data using the tampering model, we conducted backdoor attack experiments. The success rate of the backdoor attack triggered by the fault injection attack was 92%. A conventional backdoor attack was also conducted as part of a comparative experiment in which the picture with the adversarial mark was used. The success rate of the conventional attack was only 11% because the position and size of the adversarial mark varied depending on the image-capturing environment. These results demonstrate that our attack on the MIPI represents a more stable backdoor attack than the conventional method.

As mentioned previously, a DNN model can be misclassified when security measures against data tampering are not implemented on the image sensor interface. As a countermeasure against this kind of attack, a message authentication code (MAC) needs to be generated for the images on the image sensor. Then, the integrity of the image data must be evaluated before using the DNN classification system.

## Figures and Tables

**Table 1:** Experimental equipment.

Raspberry pi	Raspberry pi 4
camera module	Raspberry pi camera module v2
Oscilloscope	Keysight DSOS204A 2 GHz
Attacking FPGA	Xilinx KC705
MIPI board	Gazogiken ITL-MTR25

**Table 2:** Dataset for the experiments.

Training data	39,209
Clean training data	37,209
Poison training data	2000
Clean test data	12,630
Poison test data on the simulation	12,180
Clean test data using backdoor experiments	430 (43 class × 10)
Poison test data using backdoor experiments	420 (42 class × 10)

## Data Availability

The data are not publicly available. No data created for publication. If you have any concerns about the data, please contact the corresponding author.
